# The association between personal interest and critical thinking: a comparison between a universal (death penalty) and a local (Strait of Messina Bridge) debate

**DOI:** 10.3389/fpsyg.2026.1746352

**Published:** 2026-02-24

**Authors:** Rosa Angela Fabio, Caterina Chiara Ascone

**Affiliations:** Department of Biomedical Sciences, Dental Sciences and Morpho-functional Imaging, University of Messina, Messina, Italy

**Keywords:** controversial issues, critical thinking, ego-defensive interest, epistemic interest, motivation, personal interest, reasoning

## Abstract

**Introduction:**

This research examines how different forms of personal interest are associated with critical thinking (CT). While interest is often assumed to enhance engagement and reasoning, its role may depend not only on its intensity but also on its underlying motivational quality.

**Methods:**

Two studies investigated the relationship between personal interest and CT. In Study 1, 77 participants completed standardized CT measures and produced open-ended reasoning on two controversial issues: the death penalty (a universal topic) and the Strait of Messina Bridge (a local topic). Participants also rated their personal interest in each issue. In Study 2, 80 participants completed similar CT measures while distinguishing between different types of interest, including epistemic, ego-defensive, and identity-based interest.

**Results:**

In Study 1, a nonlinear (inverted U-shaped) relationship emerged for the local topic: both low and high levels of interest were associated with weaker CT performance, suggesting that interest intensity alone does not guarantee critical engagement. No comparable effect was found for the universal topic. Additionally, higher CT scores predicted a greater likelihood of revising one’s stance when exposed to contradictory evidence. Study 2 revealed that epistemic interest, rooted in curiosity, was positively associated with CT, whereas ego-defensive and identity-based interests showed negative associations.

**Discussion:**

Overall, these findings indicate that the quality rather than the magnitude of personal interest is more strongly linked to whether it facilitates or impairs reasoning. Interest may enhance CT when driven by epistemic curiosity, but may hinder it when tied to self-protection or identity concerns.

## Introduction

In today’s information-saturated and increasingly polarized environment, the ability to engage in critical thinking (CT) has become a fundamental cognitive skill. While critical thinking has traditionally been studied as a set of cognitive skills, growing evidence suggests that its effective deployment in real-world contexts depends not only on cognitive ability but also on motivational and affective factors. Among these, personal interest plays a particularly ambivalent role: it can foster engagement and sustained attention, but it can also bias reasoning when linked to identity or emotional investment. To clarify the conceptual foundations of this work, the following section outlines the core constructs of critical thinking and personal interest and reviews relevant motivational perspectives. Rather than merely reaffirming that critical thinking is influenced by motivation, this work seeks to clarify how different motivational dynamics shape reasoning across contexts.

## Theoretical background

### Critical thinking: conceptualization and assessment

Critical thinking (CT) has been conceptualized as a complex set of higher-order cognitive skills that enable individuals to analyze information, evaluate evidence, consider alternative perspectives, and reach justified conclusions (Facione and Facione, 2013; Halpern, 2007). Contemporary models emphasize that CT is not a single ability but a coordinated system of processes, including cognitive complexity, reflective judgment, epistemic openness, and justification of knowledge claims (Braun et al., 2020; King and Kitchener, 2004).

A key distinction in the CT literature concerns the difference between dispositional and performance-based approaches. Dispositional measures assess individuals’ self-reported tendencies toward analytical thinking, open-mindedness, and inquisitiveness (Facione et al., 1994; Sosu, 2013), whereas performance-based assessments evaluate how individuals actually reason when confronted with ill-structured problems and conflicting evidence (Braun et al., 2020; Halpern, 2013). Although dispositions are important predictors of engagement, they do not necessarily translate into high-quality reasoning in concrete contexts, particularly when issues are emotionally or ideologically charged.

For this reason, recent research has increasingly emphasized the value of performance-based assessments grounded in real-world dilemmas. Instruments such as the Critical Reasoning Assessment (CRA; Anghel et al., 2021; Fabio et al., 2025b), derived from the Reflective Judgment Model (King and Kitchener, 2004), allow for a fine-grained evaluation of multiple dimensions of reasoning, including cognitive complexity, openness, and the nature of justification. These dimensions are especially relevant for understanding how individuals reason about controversial societal issues, where uncertainty, value conflict, and competing sources of evidence are inherent. In line with motivated social cognition approaches, the present work conceptualizes critical reflection as a motivated and context-sensitive cognitive process that emerges from the interaction between individual dispositions and situational features, rather than as a purely stable trait or a purely situational response.

### Personal interest as a motivational construct

Personal interest represents a central motivational factor that is often linked to cognitive engagement and depth of processing. Interest has been shown to sustain attention, increase persistence, and promote deeper elaboration of information across learning and reasoning contexts (Hidi and Renninger, 2019; Renninger and Hidi, 2020). However, interest is not a unitary construct, and its effects on cognition depend on both its intensity and its underlying motivational orientation.

Much of the existing literature conceptualizes interest primarily in terms of intensity—how strongly an individual cares about a topic. This approach has often relied on single-item measures and has assumed a linear relationship between interest and cognitive performance (Manshaee et al., 2014; Zubaidah et al., 2018). Yet classical motivational theories, most notably the Yerkes–Dodson law (Yerkes and Dodson, 1908), suggest that performance on complex cognitive tasks follows an inverted U-shaped function: moderate levels of motivational arousal are often associated with optimal performance, whereas both low and excessively high levels can be detrimental (Broadhurst, 1959; Teigen, 1994).

Applied to critical thinking, this framework implies that moderate interest may be linked to sustained analytic engagement, whereas very low interest may be linked to disengagement and very high interest—particularly when emotionally or identity laden—may impair reflective reasoning through cognitive overload or bias. Neurocognitive evidence further supports this interpretation, indicating that optimal levels of motivational activation enhance executive control, whereas excessive activation can disrupt deliberative processing (Cools and D’Esposito, 2011; Diamond, 2013).

### Qualitative differences in interest: epistemic vs. defensive orientations

Beyond intensity, contemporary motivational research highlights the importance of distinguishing between qualitatively different types of interest. Epistemic interest, rooted in curiosity and a desire to acquire knowledge, motivates individuals to seek new information, tolerate uncertainty, and engage in reflective inquiry (Hidi and Renninger, 2019; Silvia, 2006). This form of interest has been consistently associated with deeper learning, integrative thinking, and enhanced critical reasoning (Fabio and Suriano, 2023; O'Keefe et al., 2021).

In contrast, identity-based and ego-defensive interests are oriented toward protecting one’s beliefs, values, or social identity rather than toward understanding per se. When interest is tied to self-concept or group affiliation, individuals may selectively attend to information that confirms prior beliefs and dismiss counterevidence, a pattern commonly described as motivated reasoning (Kahan, 2013). Empirical studies suggest that such defensive motivational orientations are related to narrowed cognitive focus and reduce openness, even when individuals report high engagement with the topic (Manshaee et al., 2014; Komesaroff et al., 2019).

This distinction between epistemic and defensive forms of interest is particularly relevant for reasoning about controversial social and political issues, which often activate identity concerns and emotional investment. In these contexts, high interest does not necessarily translate into high-quality critical thinking; instead, the motivational function of interest becomes a decisive factor in determining whether engagement is more likely to be accompanied by reflection or by bias.

### Interest, motivation, and critical thinking: gaps in the literature

Despite growing recognition of the motivational foundations of critical thinking, several gaps remain in the literature. First, most studies have examined interest either as a dispositional trait or as a simple intensity measure, without considering its qualitative dimensions. Second, research has predominantly relied on self-report indicators of CT rather than on performance-based reasoning tasks, limiting insight into how motivation may relate to actual reasoning processes. Third, few studies have examined how interest interacts with the contextual nature of the issue under consideration, such as differences between universal moral dilemmas and locally salient sociopolitical debates.

As a result, it remains unclear how both the intensity and the type of personal interest jointly influence critical thinking performance, cognitive flexibility, and openness to counterevidence in real-world contexts. Addressing these gaps requires an integrated approach that combines performance-based assessments of CT with a nuanced conceptualization of interest and examines reasoning across issues that vary in personal relevance and identity salience.

### The present research

The present research addresses these gaps through two complementary studies examining how personal interest—both in intensity and in motivational quality—is associated with critical thinking performance. Participants reasoned about two controversial topics that differ in scope and contextual relevance: the death penalty, a widely debated universal moral issue, and the Strait of Messina Bridge, a locally salient political and social debate.

Study 1 focuses on interest intensity, testing whether the relationship between interest and critical thinking follows a nonlinear, inverted U-shaped pattern and whether higher critical thinking is associated with greater openness to opinion change when confronted with counterarguments.

Study 2 extends this investigation by distinguishing between epistemic, identity-based, and ego-defensive forms of interest, examining their unique contributions to critical thinking performance across both dilemmas. Importantly, Study 2 was not designed to test whether motivational orientations statistically account for the interest-intensity effects observed in Study 1. Rather, it provides a complementary examination of how qualitatively different forms of interest are associated with critical thinking performance.

The distinction between the two issues is intended to capture differences in contextual scope and salience rather than a strict moral–political dichotomy, as both topics may carry ideological and identity-relevant meanings for different individuals. Together, these studies aim to clarify when and why personal interest is associated with more versus less reflective reasoning, contributing to a more integrated understanding of the motivational foundations of critical thinking. The two studies are presented in sequence below.

Beyond documenting that critical thinking is sensitive to motivational influences, the present research offers three interrelated theoretical contributions. First, it extends existing work by demonstrating that the relation between personal interest and critical thinking is not merely linear but follows a nonlinear, inverted U-shaped pattern, thereby qualifying prior assumptions that “more interest” necessarily enhances reasoning. Second, it advances motivational theorizing by distinguishing between qualitatively different forms of interest—epistemic versus identity-based and ego-defensive—and by showing that these orientations are differentially associated with critical thinking performance. Third, by comparing a universal moral issue with a locally salient sociopolitical debate, the present studies highlight the contextual conditions under which motivational factors are more strongly associated with reflective reasoning.

## Study 1

Study 1 examined the relationship between the intensity of personal interest and critical thinking performance across two types of controversial topics: a universal moral issue (the death penalty) and a locally salient political debate (the Strait of Messina Bridge). Including both a universal and a local topic allowed us to explore whether motivational and cognitive processes differ depending on the degree of personal relevance and contextual familiarity. Universal issues often elicit abstract moral reasoning, whereas local issues may evoke stronger identity-based reactions and emotional involvement, potentially biasing the reasoning process.

CT performance was assessed using the Critical Reasoning Assessment (CRA; Anghel et al., 2021; Fabio et al., 2025b), an open-ended, performance-based task grounded in the Reflective Judgment Model (King and Kitchener, 2004).

For each topic, participants reported their initial position (pro or con) and rated their personal interest, providing a measure of interest intensity. They then completed a structured reasoning task aligned with CRA dimensions, articulating and justifying their stance. Next, participants were presented with historically and scientifically accurate counterarguments that directly challenged their position and were asked whether they wished to maintain or change their opinion. This design enabled an assessment of cognitive flexibility, operationalized as openness to revising one’s viewpoint in response to valid contradictory evidence.

More specifically, the hypotheses were as follows:

*H1:* We hypothesized a nonlinear, inverted U-shaped relationship between interest intensity and CT performance on the CRA. Moderate interest was expected to be associated with greater engagement in analytic reasoning, whereas very low interest would lead to disengagement and very high interest—particularly when tied to ego-defensive motives—would promote motivated reasoning and reduce CT performance.

*H2:* We expected that higher CT performance, especially on the Openness dimension, would predict greater likelihood of revising one’s initial opinion after exposure to counterarguments. This would reflect cognitive flexibility, a hallmark of advanced critical thinking.

## Method

### Participants

A convenience sample of 77 adults was recruited to obtain a heterogeneous set of perspectives within the Italian population, although the sample is not fully representative. Recruitment occurred through two channels: online outreach (e.g., Skype) and personal networks. The online recruitment included individuals from different Italian regions via social media (e.g., Facebook, Instagram).

The final sample consisted of 50 females (64.9%) and 27 males (35.1%), aged 19–40 years (*M* = 27.83, SD = 8.04). All participants were Italian nationals: 45 (58.4%) resided in Southern Italy, 21 (27.3%) in Northern Italy, and 11 (14.3%) in Central Italy. Regarding education, 30 participants (39.0%) completed high school, whereas 47 (61.0%) held at least a bachelor’s degree. Occupational status was as follows: 44 students (57.1%), 32 employed individuals (41.6%), and 1 unemployed participant (1.3%).

### Instrument

*Critical Reasoning Assessment* (CRA; Anghel et al., 2021; Fabio et al., 2025b) was used to objectively evaluate critical thinking performance. Unlike self-report measures, the CRA focuses on actual reasoning processes, reducing self-report biases. The assessment is derived from the Reflective Judgment Model (King and Kitchener, 2004) and comprises three dilemmas related to genetics versus choice, fairness, and compassion. Participants respond to structured questions capturing their reasoning strategies and justification processes. The CRA assesses five dimensions: cognitive complexity, reasoning style, openness, nature of knowledge, and nature of justification, each rated on a 1–7 scale. Validation studies of the CRA have shown good internal consistency (*α* = 0.83) and acceptable test–retest reliability (*r* = 0.88; Anghel et al., 2021; Fabio et al., 2025b). In the present study, reliability was assessed through inter-rater agreement, which was excellent (ICC[2, 2] = 0.92), indicating highly consistent scoring across observers.

*Critical Thinking Attitude (CTA):* The Italian version of the Critical Thinking Attitude (CTA) scale, adapted from Facione et al. (1994) and Fabio et al. (2025a), was used to measure participants’ dispositions toward analytical, evaluative, and metacognitive reflection. The CTA includes 26 items across four subscales: Systematicity, Search for Truth and Openness, Analyticity, and Inquisitiveness. Responses are provided on a 5-point Likert scale (1 = strongly disagree; 5 = strongly agree), with higher scores indicating stronger critical thinking disposition. The scale has demonstrated good internal consistency (*α* = 0.89) and strong test–retest reliability (*r* = 0.96).

#### Topic-specific CRA for the death penalty and the Strait of Messina Bridge

Participants completed a topic-adapted version of the Critical Reasoning Assessment (CRA) for each issue (death penalty; Strait of Messina Bridge). For each dilemma, the five CRA dimensions—Cognitive Complexity, Reasoning Style, Openness, Nature of Knowledge, and Nature of Justification—were assessed using parallel prompts grounded in the Reflective Judgment Model (King and Kitchener, 2004).

CRA dimensions were scored on continuous 1–7 scales, with higher scores reflecting more advanced levels of reflective judgment. The examples reported below are provided for illustrative purposes only and do not imply dichotomous categorization; responses may fall at any intermediate level depending on the structure, coherence, and evidential grounding of the reasoning.

- Illustrative Examples
- Cognitive Complexity – Strait of Messina Bridge
- Low-level response (≈ 1–2)

“I think the bridge should not be built because it is useless and dangerous. It will only create problems and waste public money.”

- Mid-level response (≈ 3–4)

“The bridge could bring economic benefits, such as better connections and job opportunities, but there are also serious risks related to earthquakes and environmental damage. I am not sure which aspect should weigh more, and the issue seems complex.”

- High-level response (≈ 6–7)

“The construction of the Strait of Messina Bridge involves multiple interconnected dimensions, including economic development, environmental sustainability, seismic risk, and long-term public investment. These factors must be evaluated together, as prioritizing one inevitably affects the others. A responsible decision requires integrating evidence from engineering, environmental science, and regional planning rather than relying on a single criterion.”

- Openness – Death Penalty
- Low-level response (≈ 1–2)

“The death penalty is wrong, and there is nothing that could change my mind. Anyone who supports it is simply wrong.”

- Mid-level response (≈ 3–4)

“I am against the death penalty, but I understand that some people believe it is necessary to ensure justice or deterrence. Even so, I find it difficult to accept those arguments given the risk of irreversible mistakes.”

- High-level response (≈ 6–7)

“Although I currently oppose the death penalty, I recognize that reasonable people may hold different positions based on legal, moral, or cultural considerations. Given the complexity of the issue and the variability of evidence regarding deterrence and justice, I remain open to reconsidering my position if compelling new arguments or data were presented.”

- Nature of Justification – Death Penalty
- Low-level response (≈ 1–2)

“I know the death penalty is wrong because everyone knows it violates human rights.”

- Mid-level response (≈ 3–4)

“My view is based on what I have learned about wrongful convictions and on general human rights principles, although I am not familiar with all the legal details.”

- High-level response (≈ 6–7)

“My position is justified by empirical evidence on wrongful convictions, comparative data showing no clear deterrent effect, and ethical arguments concerning the irreversibility of capital punishment. I also consider counterarguments and evaluate their strength in light of the available evidence and the credibility of their sources.”

These examples illustrate how CRA scoring captures graded differences in reasoning quality, from concrete and unexamined responses to integrated, evidence-based, and reflective reasoning across both local and universal issues.

#### Analytic scoring rubric

Following Anghel et al. (2021) and Fabio et al. (2025a), the analytic scoring rubric (Appendix A) allowed independent scoring of the five CRA dimensions. Two trained raters evaluated participant responses. Discrepancies were resolved through discussion between raters, and consensus scores were assigned. In all cases, disagreements did not exceed one scale point on any CRA dimension. Interrater agreement approached 92% prior to consensus, with disagreements never exceeding one point. Dimensional scores were averaged and then summed to generate an overall CRA score.

### Procedure

**Figure 1:**
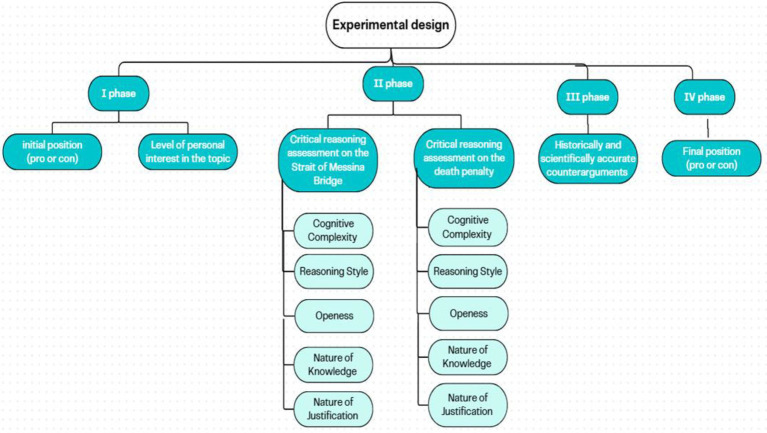
Flow of the experimental design.

Participants completed the study individually in a quiet environment. After providing informed consent, they were told that the research examined reasoning about controversial social issues (Figure 1). They were then presented with two topics in counterbalanced order: the death penalty (a universal moral dilemma) and the Strait of Messina Bridge (a locally salient sociopolitical issue). Following Anghel et al. (2021), participants were instructed to rely solely on their own knowledge and not consult external sources.

For each topic, participants first stated their initial position (pro, con, undecided) and rated their personal interest on a 1–10 scale. They then completed the topic-specific CRA, providing open-ended responses across the five dimensions of critical thinking. This phase assessed the depth, flexibility, and justification of their reasoning.

Subsequently, participants were presented with historically and scientifically accurate counterarguments that directly challenged their stance. They were asked whether they wished to maintain or revise their initial opinion, providing a behavioral measure of cognitive flexibility—defined as willingness to update one’s beliefs when confronted with strong counterevidence.

Finally, participants completed the CTA questionnaire. The full procedure lasted approximately 45–60 min.

### Statistical analysis

Analyses were conducted using IBM SPSS Statistics (Version 29; IBM Corp., 2022). Prior to hypothesis testing, data were screened for missing values, outliers, and assumption violations. Normality was evaluated using histograms, Q–Q plots, and Shapiro–Wilk tests. Linearity and homoscedasticity were assessed through residual plots. Variance inflation factors (VIF < 5) indicated no problematic multicollinearity. No extreme outliers were identified (all Cook’s distance < 1.00), and no transformations were required.

Descriptive statistics (M, SD, range) were computed for the CRA, CTA, and topic-specific CRA scores. A total of 52 written protocols were independently double coded by two trained raters using the CRA scoring rubric (Appendix A). Prior to coding, raters completed a calibration session with five practice protocols to ensure a shared understanding of the scoring criteria. Raters were blind to participants’ self-reported interest ratings, initial positions (pro/con), and opinion-change outcomes. Inter-rater reliability was excellent, with an intraclass correlation coefficient (ICC [2, 2]) of 0.92, based on a two-way random-effects model and absolute-agreement definition, indicating highly consistent scoring across observers.

Pearson correlations were used to examine associations between interest and CRA performance; point-biserial correlations were used for dichotomous variables (opinion change). Correlations were interpreted following Cohen’s (1988) guidelines.

Curvilinear relations were tested using hierarchical quadratic regressions (linear term entered at Step 1; squared term at Step 2). Improvements in model fit were evaluated using Δ*R*^2^ and associated F-tests. Assumptions were further assessed through residual diagnostics; Cook’s distances above 1.00 were considered influential.

Hypothesis 2 (critical thinking ↔ opinion change) was tested using point-biserial correlations between CRA scores (total and subscales) and post-counterargument opinion change. Alpha was set at 0.05 (two-tailed).

**Figure 2:**
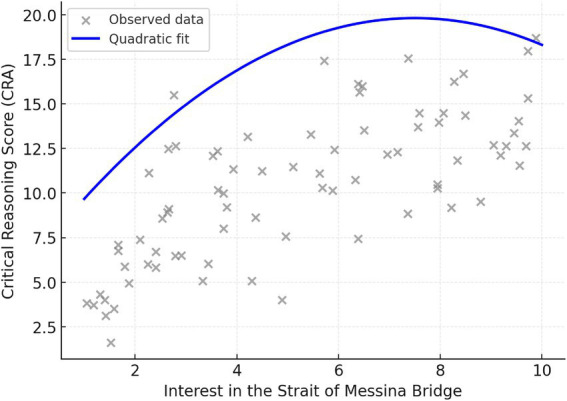
Scatterplot showing the quadratic relationship between interest in the bridge and critical reasoning performance. The inverted-U curve suggests that moderate levels of interest are associated with the highest reasoning scores, whereas both low and high levels of interest correspond to reduced critical performance.

Given the sample size, a sensitivity power analysis was conducted to determine the minimum effect size detectable with adequate power. With *N* = 77 and *α* = 0.05 (two-tailed), the study had approximately 80% power to detect correlations of *r* ≈ 0.30 or larger. Accordingly, the analyses were adequately powered to detect medium-sized effects, whereas smaller effects may not have been reliably detected (Figure 2).

## Results—study 1

Descriptive statistics for the CRA, CTA, and topic-specific CRA scores are shown in Table 1.

**Table 1:** Descriptive statistics for Critical Reasoning Assessment (CRA), Critical Thinking Attitude (CTA), and topic-specific CRA scores.

Measure	*M*	SD	Range
Critical reasoning assessment			
Total score	16.33	8.56	6–35
Cognitive complexity	2.41	3.67	1–7
Reasoning style	3.39	1.88	1–7
Openness	4.16	2.71	1–7
Nature of knowledge	3.96	1.77	1–7
Nature of justification	2.39	2.18	1–7
Critical thinking attitude			
Total score	99.71	12.99	26–130
Systematicity	35.10	6.98	9–45
Search for truth and openness	22.68	3.66	6–30
Analyticity	15.68	3.23	4–20
Inquisitiveness	26.25	4.78	7–35
CRA—death penalty			
Total score	13.50	7.50	6–35
Cognitive complexity	1.80	2.20	1–7
Reasoning style	2.50	1.60	1–7
Openness	1.50	2.00	1–7
Nature of knowledge	2.90	1.50	1–7
Nature of justification	1.80	1.80	1–7
CRA—Strait of Messina Bridge			
Total score	17.20	8.00	5–35
Cognitive complexity	2.50	3.10	1–7
Reasoning style	3.20	1.80	1–7
Openness	4.00	2.50	1–7
Nature of knowledge	3.60	1.70	1–7
Nature of justification	2.30	2.00	1–7

CRA, Critical Reasoning Assessment; CTA, Critical Thinking Attitude. CRA scores range from 5 to 35, with subscales rated on a 1–7 scale. CTA scores range from 26 to 130, with subscales scored as summed item totals according to the number of items in each dimension (Systematicity: 9 items; Search for Truth and Openness: 6 items; Analyticity: 4 items; Inquisitiveness: 7 items). The reported CRA score range reflects observed values in the present sample rather than the theoretical scoring range of the instrument. Death Penalty and Strait of Messina Bridge refer to CRA subscales applied specifically to reasoning on these topics.

Participants demonstrated a generally favorable disposition toward critical thinking (CTA total *M* = 99.71, SD = 12.99). Subscale means were consistent across domains (Systematicity, Search for Truth and Openness, Analyticity, Inquisitiveness).

For the topic-specific CRA, scores for the death penalty were lower (total *M* = 13.50, SD = 7.50), particularly regarding openness (*M* = 1.50, SD = 2.00) and cognitive complexity. In contrast, scores for the Strait of Messina Bridge were higher overall (total *M* = 17.20, SD = 8.00), including greater openness (*M* = 4.00, SD = 2.50), indicating more elaborate and flexible reasoning for the local issue.

Correlational analyses showed that CRA performance on the Strait of Messina Bridge was significantly and positively associated with overall CRA performance, *r*(77) = 0.56, *p* < 0.001, CRA on the death penalty showed only a small association with overall CRA performance, *r*(77) = 0.31, *p* < 0.05. To provide a comprehensive overview of associations among the main variables, Table 2 reports Pearson correlations among overall CRA scores, CTA, topic-specific CRA scores, and interest ratings for each dilemma. Interest ratings were examined in relation to the corresponding topic-specific CRA scores (i.e., interest in the Bridge with CRA Bridge; interest in the death penalty with CRA Death Penalty). As shown in Table 2, linear associations between interest and topic-specific critical reasoning were generally small to moderate in magnitude, supporting the need for subsequent nonlinear analyses to more accurately capture the functional form of these relationships.

**Table 2:** Correlation matrix among main study 1 variables.

Variable	1	2	3	4	5	6
1. CRA total score	—					
2. CTA total score	0.33*	—				
3. Interest—death penalty	0.21*	0.19	—			
4. Interest—Strait of Messina Bridge	0.23*	0.22	0.25	—		
5. CRA—death penalty (total)	0.31*	0.21	0.12	0.12	—	
6. CRA—Strait of Messina Bridge (total)	0.56**	0.34**	0.13	0.32*	0.31*	—

*N* = 77. Pearson correlations (two-tailed); *p < 0.05, **p < 0.01.

### Quadratic regression: interest → critical reasoning (bridge topic)

To test Hypothesis 1, a hierarchical quadratic regression examined whether interest intensity exhibited an inverted U-shaped relationship with reasoning performance on the Bridge topic. The overall model was significant: *F*(2, 75) = 5.28, *p* = 0.007, explaining about 13% of the variance in reasoning performance (*R^2^* = 0.13, adjusted *R^2^* = 0.10). Both the linear (*B* = 3.60, SE = 1.18, *p* = 0.003) and quadratic terms (*B* = −0.24, SE = 0.10, *p* = 0.020) were significant, indicating a curvilinear relationship. Residual diagnostics (Cook’s distance < 0.20) showed no assumption violations. The quadratic term significantly improved the model: Δ*R*^2^ = 0.07, *F*(1, 75) = 5.61, *p* = 0.020.

Although the estimated peak of the curve (*x* ≈ 20.3) lies beyond the observed interest range, the fitted function was consistent with an inverted U-shaped pattern, suggesting that moderate levels of interest were associated with the highest levels of critical reasoning performance (Yerkes and Dodson, 1908; Silvia, 2006). A simple linear correlation between interest in the bridge and CRA performance was significant, *r*(77) = 0.32, *p* = 0.005; however, the quadratic model provided a more accurate representation of the relationship.

### Death penalty topic

Interest in the death penalty ranged from 1 to 10 (*M* = 4.78, SD = 3.15), but showed no significant relationship with CRA performance, *r*(77) = 0.12, *p* = 0.30. Given the absence of both linear and nonlinear effects, no quadratic regression was conducted for this topic (Krosnick, 1990; Petty and Cacioppo, 1986). This pattern suggests that reasoning about the death penalty may be less sensitive to variations in self-reported interest, possibly reflecting greater attitudinal rigidity for this highly polarized moral issue.

### Critical thinking and opinion change

To test Hypothesis 2, point-biserial correlations examined associations between CRA scores and opinion change following exposure to counterarguments.

For the Strait of Messina Bridge, 29 participants (37%) revised their initial position, whereas for the death penalty only 4 participants (5%) changed their opinion, indicating markedly lower cognitive flexibility for the universal moral issue.

For the Bridge dilemma, CRA total scores were significantly associated with opinion change, *r*_pb_ (75) = 0.42, *p* < 0.001, indicating that higher critical thinking was associated with a greater likelihood of revising one’s view. The strongest association emerged for the Openness dimension (*r*_pb_ = 0.47, *p* < 0.001), suggesting that individuals who acknowledged uncertainty and alternative perspectives were more likely to revise their position.

In contrast, no significant association emerged between CRA scores and opinion change for the death penalty (*r*_pb_ = 0.08, *p* = 0.52). Taken together, these findings indicate that the link between critical thinking and behavioral flexibility was evident primarily for the locally salient issue.

It is important to note that willingness to revise one’s opinion was not intended as a direct measure of critical thinking per se, but as a behavioral indicator of cognitive flexibility in response to counterevidence. Accordingly, this outcome should be interpreted as reflecting openness to belief updating rather than as an exhaustive index of reflective reasoning.

### Study 2

Study 2 was designed to address a key limitation of Study 1. Although the first study showed that interest intensity was associated with critical thinking (CT) performance—particularly for locally relevant issues—it did not clarify why interest is associated with more versus less reasoning. Thus, Study 2 was not intended as a direct replication of the quadratic (inverted U-shaped) interest–performance effect, but rather as a complementary investigation aimed at unpacking the motivational processes that may underlie that nonlinear pattern.

Prior literature suggests that interest is not a unitary construct but reflects distinct motivational orientations that may exert divergent effects on cognition (Hidi and Renninger, 2019; Silvia, 2006). Scholars differentiate between epistemic interest, driven by curiosity and a desire to acquire knowledge, and identity-based or ego-defensive interest, which is rooted in the motivation to protect one’s beliefs, values, or self-concept (Fabio and Suriano, 2023; Kahan, 2013). Whereas epistemic interest tends to promote open-minded inquiry and reflective thought, identity-relevant motives are associated with selective information processing and motivated reasoning (Manshaee et al., 2014). For clarity, in Study 2 the term “critical thinking performance” refers exclusively to performance on the Critical Reasoning Assessment (CRA), which served as the sole measure of critical thinking in this study.

Building on these theoretical distinctions, Study 2 examined how the quality of personal interest—epistemic, identity-based, or ego-defensive—is associated with CT performance on the same two dilemmas used in Study 1. We hypothesized that epistemic interest would be positively associated with CT, whereas identity-based and ego-defensive interests would be negatively associated with CT, especially for the locally salient Messina Bridge issue, where identity concerns are more likely to be activated.

### Methods

#### Participants

A convenience sample of 80 participants was recruited within the general Italian population, although the sample cannot be considered fully representative. Recruitment was conducted through two main channels: online outreach (e.g., Skype) and personal networks. Online recruitment included individuals from various geographical regions via social media platforms such as Facebook and Instagram.

The final sample comprised 42 females (52.0%) and 38 males (48.0%), ranging in age from 19 to 40 years (*M* = 24.11, SD = 8.04). All participants were Italian nationals, with 50 (68%) residing in Southern Italy, 12 (15.5%) in Northern Italy, and 12 (15.5%) in Central Italy. Regarding education, 31 (38.8%) had completed high school, and 49 (61.2%) held at least a bachelor’s degree. In terms of occupation, 46 (57.5%) were students, 33 (41.2%) were employed, and 1 (1.3%) were unemployed.

### Instruments

The same instruments used in Study 1 were administered, along with the following interest scales.

#### Epistemic curiosity scale

The Epistemic Curiosity Scale (ECS; Litman, 2008) is a self-report instrument designed to assess epistemic curiosity, defined as the dispositional tendency to seek new information and reduce cognitive uncertainty. The scale encompasses two distinct dimensions: Interest-type epistemic curiosity (I-type EC), reflecting intrinsic motivation derived from the pleasure of discovering new knowledge, and Deprivation-type epistemic curiosity (D-type EC), representing a motivational drive to fill perceived gaps in knowledge and reduce states of uncertainty. The ECS consists of 10 items (five for each subscale), rated on a 4-point Likert scale (1 = strongly disagree, 4 = strongly agree). Subscale scores are calculated by summing responses to the corresponding items, while an overall epistemic curiosity score can be obtained by summing all items. In its original validation, the ECS demonstrated adequate internal consistency (Cronbach’s *α* = 0.79 for I-type EC; *α* = 0.84 for D-type EC) and good convergent and discriminant validity in relation to related measures of curiosity, interest, and cognitive motivation. The scale has been applied in research contexts including learning, problem solving, creativity, and motivational processes, and has been used with both university and general population samples.

#### Value–identity centrality scale

The Value–Identity Centrality Scale (Hitlin, 2003) measures the degree to which personal values are structurally integrated into an individual’s identity system. The underlying construct posits that values are not merely normative orientations or moral preferences but core components in the formation, maintenance, and regulation of the self, exerting a pervasive influence on cognitive, affective, and behavioral processes.

Respondents indicate, for a predefined set of values, the extent to which each is perceived as central and constitutive of their personal identity. Responses are typically given on a 5-point Likert scale (1 = not at all central, 5 = extremely central), allowing quantification of the identity salience assigned to each value. Scores are aggregated to produce an overall value centrality index, interpreted as an indicator of the extent to which the value system is anchored in one’s identity.

The original validation by Hitlin (2003) reported satisfactory psychometric properties, with high internal consistency (Cronbach’s *α* > 0.80) and construct validity supported by consistent correlations with convergent measures of value commitment, self-consistency, and self-reported value-congruent behaviors. The scale has been used in both sociological and psychological research, with heterogeneous samples across sociodemographic and cultural backgrounds, and in both cross-sectional and longitudinal designs. In the present study, value–identity centrality is not treated as a direct measure of interest intensity, but as an indicator of the extent to which engagement with an issue is identity-aligned. This operationalization reflects the theoretical assumption that when values central to the self are implicated, interest may serve identity-affirming or self-protective motivational functions rather than epistemic exploration.

#### Extrinsic–outward recognition subscale

The Extrinsic–Outward Recognition subscale of the Work Preference Inventory (WPI; Amabile et al., 1994) was administered to assess participants’ motivation related to external approval and acknowledgment. This subscale includes items reflecting the extent to which individuals value recognition, praise, or esteem from others as a source of motivation. Items were rated on a 4-point Likert scale (1 = strongly disagree, 4 = strongly agree), without a neutral midpoint to minimize central-tendency bias. Previous validation studies of the WPI have reported satisfactory internal consistency (*α* ≈ 0.70–0.85 for this subscale) and evidence of convergent validity with other measures of extrinsic motivation and work engagement (Amabile et al., 1994). In the present study, only this subscale was employed to reduce participant burden while maintaining a reliable indicator of ego-defensive or recognition-based motivational orientation.

In the present study, value–identity centrality was used as an indicator of identity-based interest insofar as it captures the degree to which specific values related to the debated issues are integrated into participants’ self-concept. When an issue is closely tied to core personal values, engagement with that issue is more likely to serve identity-affirming functions rather than epistemic exploration. This operationalization is consistent with prior work conceptualizing identity-based motivation as the extent to which attitudes and values are central to the self (Hitlin, 2003; Kahan, 2013).

### Statistical analysis

All analyses were conducted using IBM SPSS Statistics (Version 29; IBM Corp., 2022). Data were screened for missing values, outliers, and assumption violations. Shapiro–Wilk tests and Q–Q plots indicated no substantial departures from normality; scatterplots of standardized residuals confirmed linearity and homoscedasticity; and Cook’s distances were all below 1.00.

Descriptive statistics and zero-order correlations were computed for epistemic, identity-based, and ego-defensive interest and CRA performance in both dilemmas. Pearson correlations were interpreted using Cohen’s (1988) guidelines.

To assess the unique contributions of each type of interest to CRA performance, two separate standard multiple regression analyses were conducted—one for the Bridge dilemma and one for the Death Penalty dilemma. In each model, epistemic, identity-based, and ego-defensive interest scores were entered simultaneously as predictors, with CT performance as the dependent variable. Statistical significance was set at *α* = 0.05 (two-tailed) for all tests. Given the sample size, a sensitivity power analysis was conducted to determine the minimum effect size detectable with adequate power. With *N* = 80 and *α* = 0.05 (two-tailed), the study had approximately 80% power to detect correlations of *r* ≈ 0.28 or larger. Accordingly, the analyses were adequately powered to detect medium-sized effects, whereas smaller effects may not have been reliably detected.

## Results—study 2

In Study 2, critical thinking performance was operationalized exclusively through CRA scores, allowing direct comparability with Study 1. Pearson correlations indicated that epistemic interest was positively and significantly associated with CRA performance for both the Bridge dilemma, *r*(78) = 0.54, *p* = 0.003, and the Death Penalty dilemma, *r*(78) = 0.46, *p* = 0.004 (Table 3). Identity-based interest showed small, non-significant negative correlations with CRA performance in both dilemmas, *r* = −0.29 and *r* = −0.20, respectively (*ps* > 0.05). Ego-defensive interest was also negatively associated with CRA performance, with a moderate but non-significant correlation for the Death Penalty dilemma, *r*(78) = −0.38, *p* = 0.060, and a smaller, non-significant correlation for the Bridge dilemma, *r*(78) = −0.30, *p* = 0.11.

**Table 3:** Pearson correlations among study variables.

Variable	1	2	3	4	5
1. Epistemic interest	—	0.18	−0.10	0.54**	0.46**
2. Identity-based		—	−0.07	−0.29	−0.20
3. Ego-defensive			—	−0.30	−0.38
4. CT—Bridge dilemma				—	0.72**
5. CT—Death penalty					—

**p < 0.01.

Two multiple regressions assessed the unique predictive value of each interest type.

For the *Bridge dilemma*, the model was significant, *R*^2^ = 0.31, *p* < 0.001. Epistemic interest positively predicted CT (*β* = 0.57, *p* < 0.001), whereas ego-defensive interest negatively predicted CT (*β* = −0.21, *p* = 0.041). Identity-based interest was not a significant predictor (*β* = −0.13, *p* = 0.201).

For the *Death penalty dilemma*, the model was also significant, R2 = 0.23, *p* < 0.001. Epistemic interest again positively predicted CT (*β* = 0.43, *p* < 0.001), and ego-defensive interest negatively predicted CT (*β* = −0.29, *p* = 0.009); identity-based interest was not significant (*β* = −0.04, *p* = 0.713). Diagnostic checks indicated no problematic multicollinearity (all VIFs < 5), suggesting that the observed effects reflected distinct motivational contributions rather than shared variance among predictors (Table 4).

**Table 4:** Results of multiple regression analyses predicting critical thinking performance on the Bridge dilemma and Death penalty tasks from three types of personal interest.

Predictors	*β* (Bridge dilemma)	*t*	*p*	*β* (Death penalty)	*t*	*p*
Epistemic interest	0.57	5.41	<0.001	0.43	3.91	<0.001
Identity-based	−0.13	−1.29	0.201	−0.04	−0.37	0.713
Ego-defensive	−0.21	−2.10	0.041	−0.29	−2.68	0.009
*R* ^2^	0.31			0.23		

### Summary of study 2 results

Although identity-based interest did not emerge as a significant predictor, the direction of its associations with CRA performance was consistently negative. Ego-defensive interest showed significant negative associations in both regression models, whereas epistemic interest showed robust positive associations across dilemmas. These results indicate that the quality of personal interest, rather than its mere presence, is systematically related to critical thinking performance.

## Discussion

The present research examined how both the intensity and the motivational quality of personal interest are associated with critical thinking (CT) performance across two controversial societal debates: a universal moral dilemma (death penalty) and a locally salient political issue (Strait of Messina Bridge). Drawing on the Yerkes–Dodson law (Yerkes and Dodson, 1908) and contemporary dual-process theories (Evans and Stanovich, 2013), we proposed that moderate levels of interest would be associated with higher CT by coinciding with greater engagement in effortful, analytical System 2 processes, whereas both low and high levels—especially when linked to ego-defensive or identity-based motives—might be associated with lower performance through disengagement or motivated reasoning (Fabio and Caprì, 2023; Kahan, 2013; Silvia, 2006). Given the correlational nature of both studies, the present findings should be interpreted as evidence of association rather than causation, and alternative explanations cannot be ruled out.

### Summary of findings

Overall, the findings of the two studies converge in showing that both the intensity and the motivational orientation of interest are relevant for understanding variability in critical thinking performance, with effects that depend on issue context.

Study 1 supported the hypothesized nonlinear relationship between interest intensity and CT performance for the locally salient Bridge dilemma, revealing an inverted U-shaped pattern. This pattern suggests that moderate interest may be optimal for reflective reasoning, whereas very high interest may coincide with emotional or identity involvement that constrains analytical processing. Because motivational mechanisms were not directly measured, this interpretation remains theoretical. No comparable relationship emerged for the death penalty, suggesting that highly polarized moral issues may elicit rigid attitudes that weaken the link between interest and reasoning performance (Krosnick, 1990; Petty and Cacioppo, 1986). Additionally, higher CRA scores—particularly on the Openness dimension—were associated with greater willingness to revise one’s opinion, but only in the locally salient condition.

Study 2 further clarified that interest quality is more informative than interest intensity alone. Epistemic interest, reflecting curiosity and a desire for understanding (Hidi and Renninger, 2019; Silvia, 2006), was positively associated with CT across both dilemmas. In contrast, identity-based and ego-defensive interests tended to show negative associations with CT. Notably, ego-defensive interest emerged as a significant negative predictor even when controlling for other motivational orientations, indicating a specific association with reduced openness to counterevidence (Fabio and Suriano, 2023; Fabio and Suriano, 2025; Manshaee et al., 2014).

Although some effects were consistent across topics, others were context dependent. Nonlinear effects of interest intensity and the association between CT and opinion change emerged only for the locally salient issue, suggesting that contextual relevance and attitudinal rigidity condition how motivational factors shape reasoning.

### Theoretical implications

The present research advances theorizing on CT and motivation by moving beyond the assumption that interest uniformly enhances reasoning quality. Instead, the findings indicate that the effects of interest depend jointly on its intensity, its motivational orientation, and the contextual characteristics of the issue under consideration.

A first contribution lies in demonstrating that the relationship between interest intensity and CT is not necessarily linear. Extending classical motivational models such as the Yerkes–Dodson law to complex reasoning, Study 1 shows that both insufficient and excessive interest may be associated with lower reflective performance. These findings challenge implicit assumptions in the CT literature that increasing engagement alone is sufficient to foster higher-order reasoning.

A second contribution concerns the differentiation of motivational orientations underlying interest. Study 2 demonstrates that epistemic interest is consistently associated with higher CT performance, whereas ego-defensive motivation is associated with lower performance. This distinction helps explain why high engagement may sometimes coexist with selective or biased reasoning, particularly in controversial contexts.

A third contribution highlights the role of contextual relevance. By comparing a universal moral dilemma with a locally salient sociopolitical issue, the present research shows that motivational effects on CT are not uniform across contexts. Issues characterized by strong moral polarization may constrain reasoning processes regardless of individual differences in interest, whereas locally relevant issues may allow greater variability in reflective engagement.

Taken together, these contributions support a view of CT as a motivated cognitive activity whose quality depends on both the nature of the task and the underlying motivational orientation. This integrative perspective refines existing models by jointly considering motivational intensity, motivational quality, and contextual relevance.

### Practical implications

These findings have clear implications for educational and civic contexts. Instructional strategies that foster epistemic curiosity—such as encouraging perspective-taking and framing issues as complex and open-ended—may support higher-quality reasoning (O'Keefe et al., 2021; Tong et al., 2023). By contrast, approaches that primarily activate identity-based motivations may increase engagement while inadvertently constraining analytical depth.

For interventions aimed at reducing polarization, the present results suggest that promoting the quality of interest—rather than merely increasing engagement intensity—may be critical for supporting reflective and open-minded reasoning.

### Limitations and future directions

Several limitations should be acknowledged. First, self-reported interest may not fully capture underlying motivational states, particularly for emotionally charged issues. Future research could complement self-reports with behavioral or physiological measures (Cools and D’Esposito, 2011; Diamond, 2013). A related limitation concerns the CRA itself. Although the CRA provides a performance-based measure of reasoning, it also reflects expressive and linguistic components that may partially influence scores independently of reasoning quality. Future studies should therefore control for response length, linguistic complexity, and educational background.

Second, sample size limits the detection of small effects. Some non-significant findings, particularly for identity-based and ego-defensive interests, may reflect limited statistical power rather than the absence of meaningful associations. Moreover, several effects were close to conventional significance thresholds and should be interpreted cautiously.

Third, the operationalization of cognitive flexibility through opinion change may reflect multiple underlying processes. Revising weakly held beliefs may differ qualitatively from revising strongly held convictions. Future research should incorporate independent measures of epistemic certainty and confidence.

Finally, the cross-sectional design precludes causal inference. Experimental manipulations of interest type and intensity are needed to test causal pathways. An alternative interpretation of the nonlinear pattern observed for the Bridge dilemma is that both moderate interest and higher CRA scores reflect greater tolerance for epistemic uncertainty rather than interest intensity per se. The absence of a similar pattern for the death penalty supports this possibility and highlights directions for future research.

### Conclusion

This research advances understanding of how personal interest relates to critical thinking by demonstrating that both interest intensity and motivational quality matter. Through two studies, epistemically oriented interest was most consistently associated with higher-quality reasoning, particularly in locally salient debates. By contrast, interest oriented toward identity defense was associated with reduced openness to counterevidence. These findings suggest that promoting effective critical thinking requires fostering motivational orientations that support reflective inquiry, rather than simply increasing engagement.

## Data Availability

The original contributions presented in the study are included in the article/Supplementary material, further inquiries can be directed to the corresponding author.
