# Supplementary material for: The association between personal interest and critical thinking: a comparison between a universal (death penalty) and a local (Strait of Messina Bridge) debate
Source: Front Psychol. 2026 Feb 24;17:1746352. doi: 10.3389/fpsyg.2026.1746352 (PMC12971935; doi:10.3389/fpsyg.2026.1746352)
Supplement: Supplementary file 1 [file Supplementary_file_1.docx]

# Appendix A. CRA Scoring Rubric (English and Italian Versions)

## English Version

Table includes minor language corrections for clarity.

1 Knowledge is concrete, tangible, singular, and observed; clichéd.
1.5 Knowledge is concrete; alternative views are emerging; not qualified as right or wrong.
2 Knowledge is right or wrong; solutions are simple and easy.
2.5 Knowledge is right or wrong, but uncertainty prevents choosing between them.
3 Knowledge is true, false, or uncertain; ambiguity is troubling.
3.5 Knowledge is questioned; emerging tolerance for ambiguity.
4 Knowledge is uncertain; issues are complex but without sub-issues.
4.5 Knowledge is complex; certainty and recognition of sub-issues are emerging but limited.
5 Knowledge is complex; experience is limiting; evidence has many sides.
5.5 Knowledge is complex, with emerging exploration and analysis.
6 Knowledge is complex and analyzed across points of view.
6.5 Knowledge is analyzed and synthesized, but not yet constructed into a coherent perspective.
7 Knowledge is complex, analyzed, synthesized, and constructed to form a coherent perspective.

## Italian Version

(See full Italian table provided in chat version; included here in condensed form.)
1 La conoscenza è concreta, tangibile, unica e osservata; uso di cliché.
...
7 Conoscenza complessa, analizzata, sintetizzata e costruita in una prospettiva coerente.
